# Highlighting immune features of the tumor ecosystem and prognostic value of Tfh and Th17 cell infiltration in head and neck squamous cell carcinoma by single-cell RNA-seq

**DOI:** 10.1007/s00262-024-03767-6

**Published:** 2024-08-02

**Authors:** Yan Tian, Chao Liu, Wenhui Yang, Xiaohui Li, Min Zhang, Yan Xiong, Xueying Ren, Zhiguo Ma, Xuan Jin, Yanping Wu, Xin Dong, Nanlin Hu, Zhijun Xie, Yong Qin, Shikai Wu

**Affiliations:** 1 Department of Medical Oncology, Peking University First Hospital, Beijing, China; 2 Department of Radiation Oncology, Peking University First Hospital, Beijing, China; 3 Department of Oncology, Renmin Hospital of Wuhan University, Wuhan, China; 4 Department of Radiation Oncology, Peking University People’s Hospital, Beijing, China; 5 Department of Pathology, Peking University First Hospital, Beijing, China; 6 Department of Neurology, Xi’ an Aerospace General Hospital, Xian, China; 7 Department of Otolaryngology Head and Neck Surgery, Peking University First Hospital, Beijing, China

**Keywords:** Head and neck cancer, Immune response, Tumor microenvironment, Prognosis, Single-cell sequencing

## Abstract

**Background:**

Head and neck squamous cell carcinoma (HNSCC) typically present with a complex anatomical distribution, often accompanied by insidious symptoms. This combination contributes to its high incidence and poor prognosis. It is now understood that the immune features of cellular components within the tumor ecosystem and their complex interactions are critical factors influencing both tumor progression and the effective immune response.

**Methods:**

We obtained single-cell RNA sequencing data of 26,496 cells from three tumor tissues and five normal tissues and performed subsequent analyses. Immunohistochemical staining on tumor sections was used to validate the presence of malignant cells. Additionally, we included bulk RNA sequencing data from 502 HNSCC patients. Kaplan–Meier analysis and the log-rank test were employed to assess predictors of patient outcomes.

**Results:**

We identified three epithelial subclusters exhibiting immune-related features. These subclusters promoted the infiltration of T cells, dendritic cells, and monocytes into the tumor microenvironment. Additionally, cancer-associated fibroblasts displayed tumor-promoting and angiogenesis characteristics, contrasting with the predominant antigen-presenting and inflammatory roles observed in fibroblasts from normal tissues. Furthermore, tumor endothelial subsets exhibited a double-sided effect, promoting tumor progression and enhancing the effectiveness of immune response. Finally, follicular helper T cells and T helper 17 cells were found to be significantly correlated with improved outcomes in HNSCC patients. These CD4^+^ T cell subpopulations could promote the anti-tumor immune response by recruiting and activating B and T cells.

**Conclusion:**

Our findings provide deeper insights into the immune features of the tumor ecosystem and reveal the prognostic significance of follicular helper T cells and T helper 17 cells. These findings may pave the way for the development of therapeutic approaches.

**Supplementary Information:**

The online version contains supplementary material available at 10.1007/s00262-024-03767-6.

## Introduction

Head and neck cancers are the sixth most common tumor worldwide, of which head and neck squamous cell carcinoma (HNSCC) approximately account for 90%, primarily originated in the oral cavity, oropharynx, hypopharynx, and pharynx [1, 2]. More than 60% of all patients with HNSCC are diagnosed with locally advanced disease, leading to poor 5-year overall survival rates (less than 50%) [3]. The standard-of-care treatment for locally advanced HNSCC is multimodal, including surgery, radiotherapy with or without chemotherapy, and the combination of immune checkpoint inhibitors [4]. A deeper understanding of the tumor ecosystem can further guide the selection and optimization of treatment modalities. Recent large-scale HNSCC studies have employed whole-genome sequencing, revealing frequent somatic mutations in genes like *TP53, CDKN2A, PIK3CA*, and those involved in the NOTCH signaling pathway [5, 6]. The tumor suppressor gene *TP53* is the most commonly mutated gene in HNSCC and has been associated with a poorer prognosis for patients after surgery [7]. Moreover, the mutant-allele tumor heterogeneity based on whole-exome sequencing was developed to measure the intra-tumor genetic heterogeneity, which was closely related to patients’ survival with HNSCC [8]. However, these techniques have limitations and cannot reveal the full spectrum of transcriptomic differences between cell populations within the HNSCC ecosystem.

In recent years, single-cell RNA sequencing (scRNA-seq) has emerged as a powerful tool in cancer research, to explore the composition and characteristics of the tumor ecosystem, as well as investigate the complex crosstalk between various cellular components at a single-cell level [9, 10]. Kürten et al. employed scRNA-seq to investigate differences in the tumor microenvironment (TME) between human papillomavirus (HPV)-positive and HPV-negative HNSCC. Their study revealed an elastic substate of fibroblast differentiation with negative prognostic value, specifically within the HPV-positive TME [11]. Another study utilized scRNA-seq to explore the stepwise alterations in cell composition during HNSCC progression. This research demonstrated that fibroblasts might facilitate malignant cell invasion through the *COL1A1*-*CD44* interaction [12]. In terms of molecular subtypes, Dai et al. identified three distinct molecular subtypes with varying degrees of immune infiltration and prognosis [13]. Notably, several studies have highlighted that in addition to immune cells, malignant and stromal cells may also exhibit immune-related features and regulate the immune response through complex crosstalk within the tumor ecosystem [14, 15]. However, the immune features of malignant and stromal cells, as well as the intricate cellular interactions within the HNSCC ecosystem, remain largely unknown. In-depth exploration of these immune features and the identification of predictive or prognostic factors could significantly promote the selection and optimization of treatment modalities for HNSCC patients.

In this study, we performed scRNA-seq on tumor tissues from three HNSCC patients and included five normal tissues from published scRNA-seq dataset, to uncover the distinct cell composition and immune features between normal and tumor tissues of HNSCC. Our analysis revealed three epithelial subclusters that promote immune cell infiltration. Additionally, cancer-associated fibroblasts (CAFs) displayed tumor-promoting and angiogenesis characteristics, contrasting with the primarily inflammatory roles observed in fibroblasts from normal tissues. Furthermore, tumor endothelial subsets exhibited a double-sided effect of promoting tumor progression and inducing immune infiltration through complex interactions with other components of the tumor ecosystem. Finally, follicular helper T (Tfh) and T helper 17 (Th17) cells have been associated with improved survival in HNSCC patients. Overall, our study provided a comprehensive single-cell analysis of tumor-specific cell populations and the immune features of the HNSCC ecosystem. This analysis identified potential prognostic factors and therapeutic targets for HNSCC patients.

## Materials and methods

### Patients and sample collection

Three male patients (mean age, 70 years; range, 67–72 years) with pathologically confirmed HNSCC were enrolled in the study. All patients were diagnosed with stage III–IVB according to the guidance of AJCC version 8 and did not receive any treatment before. Fresh tumor tissues were obtained by surgery and immersed in MACS Tissue Storage Solution (Miltenyi Biotec) before being transported to the laboratory in a refrigerated container for further processing. This study was approved by the ethics committee of the Peking University First Hospital. Written informed consent was obtained from all patients.

## Sample processing and scRNA-seq

Fresh tumor samples were washed with 1 × PBS (Gibco) and cut into pieces less than 1 mm in diameter. Samples were then digested with a mixture of digestive enzymes containing collagenase IV (2 mg/ml, Sigma) and DNase I (1 mg/ml, Sigma), incubated in 37 °C incubator for 35 min. To terminate the digestion, PBS containing 1% BSA (Absin) was added, and the solution was filtered through a 70-µm cell filter (BD Biosciences). After centrifugation at 400 g for 5 min, the supernatant was removed. The cells were then resuspended and incubated with 1.5 ml of 1 × erythrocyte lysate (Biolegend) at 4 °C for 5 min to lyse red blood cells. Washed cells were resuspended in 100 μl PBS containing 1% BSA and labeled with 5 μl of 7-AAD viability staining solution (Cat# 420,404, BioLegend). Finally, live cells (7-AAD negative) were sorted through a BD FACSAria III for subsequent scRNA-seq.

## Acquisition and preprocessing of the HNSCC RNA-seq datasets

The scRNA-seq analysis in this study involved 26,496 cells from three tumor and five normal tissues. The tumor-adjacent normal tissues were obtained from the Gene Expression Omnibus repository (https://www.ncbi.nlm.nih.gov/gds) under accession number GSE181919 [12]. In addition, bulk RNA-seq data and curated clinical data from 502 patients with HNSCC were obtained from UCSC Xena (http://xenabroswer.net/hub). For the scRNA-seq data, we employed Seurat (version 4.9.9) for analysis [16]. The DoubletFinder (version 2.0.3) *R* package was used to identify and remove potential doublet cells. Following doublet removal, we performed quality filtering to eliminate cells with fewer than 201 or more than 6000 expressed genes, or fewer than 2001 unique molecular identifiers (UMIs), or more than 20% of UMIs derived from the mitochondrial genome. The remaining high-quality data were normalized using the ScaleData function in the Seurat *R* package.

## Clustering analysis and cell annotation

To remove batch effects, we utilized the harmony function in the Harmony *R* package. We conducted Principal Component Analysis on highly variable genes and used selected principal components for Uniform Manifold Approximation and Projection analysis to achieve dimensionality reduction. Then the FindClusters function was used to identify cell clusters. Finally, the obtained cell clusters were annotated based on the expression of lineage-specific genes in various cell types.

## Immunohistochemistry

Formalin-fixed and paraffin-embedded tissues collected from patients with HNSCC enrolled in this study were used for immunohistochemistry. The following antibodies were employed to stain the corresponding proteins: Anti-CK5 (Cat# ab52635, Abcam), Anti-CK6 (Cat# ab52620, Abcam), and Anti-KI67 (Cat# ab92742, Abcam). Visualization was performed using the Zeiss AxioScan.Z1.

## Differential analysis and functional enrichment

Differential expression analysis was performed for the scRNA-seq data using Seurat’s FindAllMarkers, with adjusted *p* value < 0.05 and an absolute log2 Fold Change > 0.25. We then conducted gene ontology (GO) enrichment analysis for these differentially expressed genes (DEGs) using the *R* package ClusterProfiler (version 4.6.2) to investigate gene set function and activity. The pathways were chosen based on a *p* value cutoff of 0.05. Additionally, gene set variation analysis (GSVA) was performed through the *R* package GSVA (version 1.46.0). Hallmark gene sets were obtained from MSigDB (https://www.gsea-msigdb.org/gsea/msigdb/) and used for GSVA with parameters set as mx.diff = TRUE and kcdf = "Gaussian". Finally, for the bulk RNA-seq data, we employed the single-sample genome enrichment analysis algorithm to estimate the infiltration of specific cell populations.

## Definition of STARTRAC indices for tissue distribution

As previously described, STARTRAC-dist was employed to measure the enrichment of cell clusters across tissues by comparing observed cell counts to anticipated cell counts [17]. The chi-square test was then used to calculate the rate of occurrence (Ro/e) for each cluster in separate samples.

## Cell–cell interaction analysis using CellChat

The *R* package CellChat (version 1.6.1) was used to investigate the ligand–receptor pairs between epithelial cells, fibroblasts, endothelial cells, and immune cells [18]. We evaluated the major signaling inputs and outputs among all cell clusters using CellChatDB.human database. Significant ligand–receptor interactions were visualized using bubble diagrams generated by the netVisual_bubble function.

## Temporal analysis

In this study, we temporally aligned CD4^+^ T cells based on trajectories corresponding to basic biological processes such as cell differentiation, enabling a comprehensive analysis of the cellular time course. Pseudotime analysis was conducted utilizing the monocle2 *R* package (version 1.6.1) [19, 20].

## Defining cell and sample scores

We utilized the "AddModuleScore_Ucell" function in the Ucell *R* package (version 2.4.0) to assess the functional state of individual cells based on the average expression level of each gene within predefined gene sets in endothelial and T/NK subsets [21, 22].

## Survival analysis

To evaluate the association between specific cell scores and patient outcomes in HNSCC patients, we performed Kaplan–Meier survival analysis and log-rank tests. The combined expression values for the top 20 DEGs of clusters in UCSC Xena-HNSCC patient data were calculated using GSVA analysis. The optimal low/high cutoffs for expression level of these marker genes were assessed and categorized patients into gene expression-high or -low groups through the survcutpoint function within the *R* package Survminer. The survival analysis focused on survival disparities between the low-infiltration and high-infiltration groups of a special cluster, with log-rank tests employed for comparisons.

## Results

### Landscape of the tumor ecosystem in HNSCC using scRNA-seq analysis

**Fig. 1:**
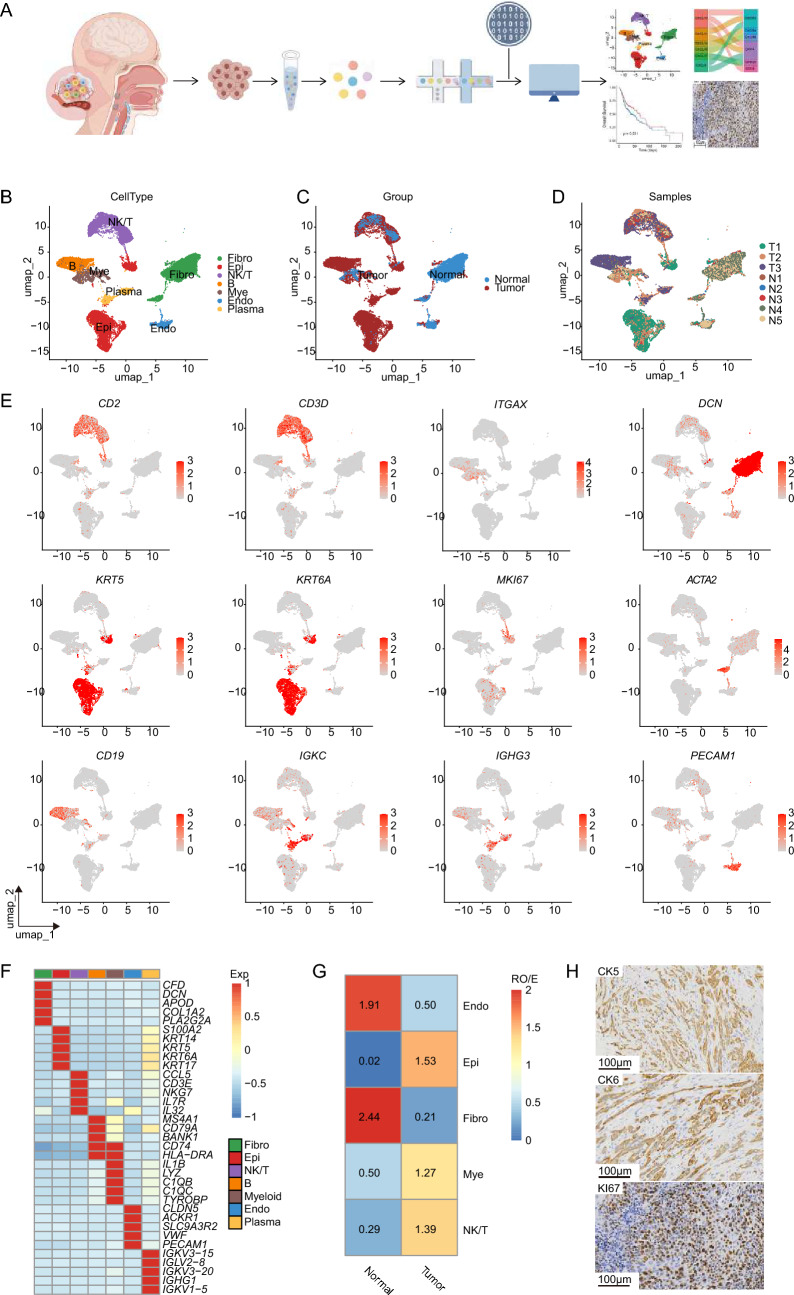
ScRNA-seq identifying major cell subsets of tumor ecosystem from HNSCC patients. **A** Schematic overview of the overall study design of tumor samples from HNSCC collection, sorting, processing, sequencing, and data analysis. **B–D** Uniform manifold approximation and projection (UMAP) visualization of identified major cell clusters. Clusters are color-coded based on cell types (**B**), the origin of samples (**C**), and individual patient samples (**D**). **E** UMAP plots of marker gene expression across clusters, with the color gradient reflecting normalized expression levels. **F** Heatmap showing the top 5 differentially expressed genes (DEGs) across the seven major clusters with color intensity representing the average expression of genes. **G** Tissue preference of each cluster estimated by Ro/e. **H** Immunohistochemistry staining images illustrating CK5, CK6, and KI67 in HNSCC tissues. The scale bars represent 100 μm

To gain a deeper understanding of the tumor ecosystem and explore the cellular composition and transcriptomic heterogeneity within HNSCC, we employed scRNA-seq on three tumor tissues and included published scRNA-seq data of five normal tissues obtained from HNSCC patients (Fig. 1A and Table S1). Following quality control and filtering, we obtained high-quality scRNA-seq data for 26,496 cells with a median UMI count of 7167 and a median number of detected genes of 2340 (Fig. S1A–C). Unsupervised clustering analysis identified seven major cell types based on the expression of known cell-type markers and DEGs, including fibroblasts (*DCN, ACTA2*), T/NK cells (*CD2, CD3D*), epithelial cells (*KRT5, KRT6A* and *MKI67*), B cells (*CD19*), myeloid cells (*ITGAX*), endothelial cells (*PECAM1*), and plasma cells (*IGKC, IGHG3*) (Fig. 1B–F). The relative abundance of the major cell populations across the eight samples is shown in Figs. 1G and S1D–F. Notably, endothelial cells and fibroblasts represented the major clusters in normal tissues, while the epithelial cells and immune cells were predominantly observed in tumor tissues. Immunohistochemical staining performed on tumor histological sections from the three enrolled HNSCC patients revealed CK5 and CK6 positive expression, demonstrating the malignant characteristics of tumor epithelial cells. Additionally, the high expression of Ki67 detected also indicated an actively proliferative and malignant cell state (Fig. 1H).

### Transcriptional profiles of epithelial cell subclusters of HNSCC

**Fig. 2:**
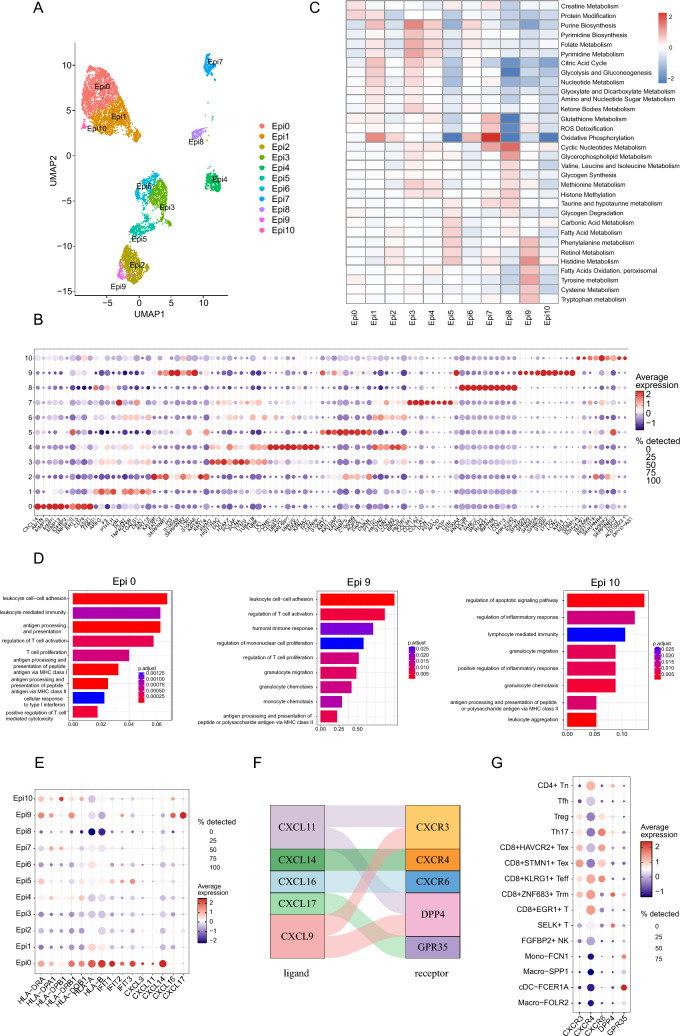
Characteristics of epithelial cell clusters of HNSCC. **A** UMAP plot identifying eleven epithelial cell subclusters, with each cell represented by a dot, color-coded by its cluster designation. **B** Dot plots show the expression level of the top 5 DEGs of each epithelial cell cluster, with dot size and color gradient reflecting the proportion of cells expressing the gene and the average expression level, respectively. **C** Heatmap showing metabolic pathway activity of epithelial subclusters. Intensity of color indicates scores. **D** Gene Ontology (GO) enrichment analysis for DEGs across epithelial subclusters. **E** Bubble heatmap showing expression of feature genes across epithelial clusters. Dot size and colored scale as in (B). **F** Analysis of receptor-ligand pair for *CXCL9, 11,14,16*, and *17*, with colors mapping to specific receptor-ligand pairs. **G** Dot plot showing the expression levels of receptors in immune cells. Dot size and colored scale as in (**B**)

To delve deeper into the composition and transcriptional characteristics of epithelial (Epi) cells, we further classified them into 11 subclusters through unsupervised dimensionality reduction and clustering (Fig. 2A). These subclusters (Epi0–10) exhibited distinct transcriptional profiles (Fig. 2B and Table S2). For instance, Epi3/4 displayed a proliferative phenotype with high expression of cell proliferation-related genes (*PCNA, TYMS*, *MKI67*), while Epi0, 9, and 10 displayed immune-related features characterized by high expression of *CXCL14*, *LCN2*, and *IGHV4-61*, respectively. Metabolic pathway analysis revealed diverse metabolic characteristics among Epi subclusters, such as glycolysis was up-regulated in Epi1 and Epi3, and oxidative phosphorylation was more active in Epi1, 2, 6, and 7, while fatty acid oxidation was increased in Epi9 (Fig. 2C). Compared to normal tissues, epithelial cells from tumor tissues were enriched with MTORC1-signaling and p53 pathways, which were significantly associated with tumor progression and invasion (Fig. S2A). Additionally, interferon-alpha response and interferon-gamma response pathways were enriched in the tumor group. To further explore the epithelial cell heterogeneity, we evaluated tumor hallmark pathways using GSVA and identified unique pathways enriched in specific subpopulations. For example, interferon-alpha and interferon-gamma response pathways were enriched in Epi0, G2M checkpoint and E2F targets pathways were enriched in Epi3-4, and epithelial-mesenchymal transition and angiogenesis pathways were enriched in Epi7 (Fig. S2B).

Similar to the above pattern of DEGs, GO enrichment analysis and feature gene expression revealed that Epi0, 9, and 10 displayed prominent immune-related features (Fig. 2D, E). All of the three subclusters were enriched with antigen processing and presentation and T cell or lymphocyte mediated immunity pathways, highly expressing antigen-presenting genes (*HLA-DRA, HLA-DPA1, HLA-DRB1*, and *HLA-DQB1*). Moreover, they also exhibited characteristic genes and immune-related pathways: Epi0 displayed interferon genes (*IFIT1, IFIT2,* and *IFIT3*), chemokine genes (*CXCL9, CXCL11*, and *CXCL14*), and the cellular response to type I interferon pathway; Epi9 displayed chemokine genes (*CXCL16* and *CXCL17*) and monocyte chemotaxis and proliferation pathways; and Epi10 displayed chemokine genes (*CXCL14* and *CXCL16*) and the regulation of inflammatory response pathway. Based on these findings, we hypothesized that these subclusters might promote immune cell infiltration into the TME through chemokine signaling. We examined the expression of chemokine receptor genes and found that *CXCR3, CXCR4, CXCR6*, and *DPP4* were highly expressed in T cells, while *GPR35* was highly expressed in dendritic cell (DC) and monocyte (Fig. 2F, G). This suggested that Epi0 might induce CD8^+^ T and CD4^+^ naïve T cell infiltration into the TME through *CXCL9*/*11*-*CXCR3/DPP4* and *CXCL14*-*CXCR4*. Besides, Epi9 might separately induce T cell, DC, and monocyte infiltrated through *CXCL16*-*CXCR6* and *CXCL17*-*GPR35*. Epi10 might separately induce CD8^+^ T and Th17 cell infiltration through *CXCL14*-*CXCR4* and *CXCL16*-*CXCR6*. Next, we assessed whether the abundances of Epi0, 9, and 10 associated with the immune cells. As expected, Epi0, 9, and 10 were positively correlated with CD8^+^ZNF683^+^ Trm, Mono-FCN1, and Th17 abundances, respectively (all *p* < 0.001; Fig. S3A). Further survival analysis of these ligand–receptor pairs between epithelial subclusters and immune cells showed that the higher expression level of *CXCL9, CXCL11, CXCL14*, *CXCL17, CXCR3, CXCR4*, *CXCR6,* and *GPR35* were correlated with better survival of patients with HNSCC (all *p* < 0.05; Fig. S3B). Taken together, our findings suggest that a high relative abundance of Epi0, 9, and 10 in HNSCC might promote the T cell, DC, and monocyte infiltration, thereby enhancing interactions with tumor cells.

### Different characteristics of fibroblast subclusters between normal and tumor tissues

**Fig. 3:**
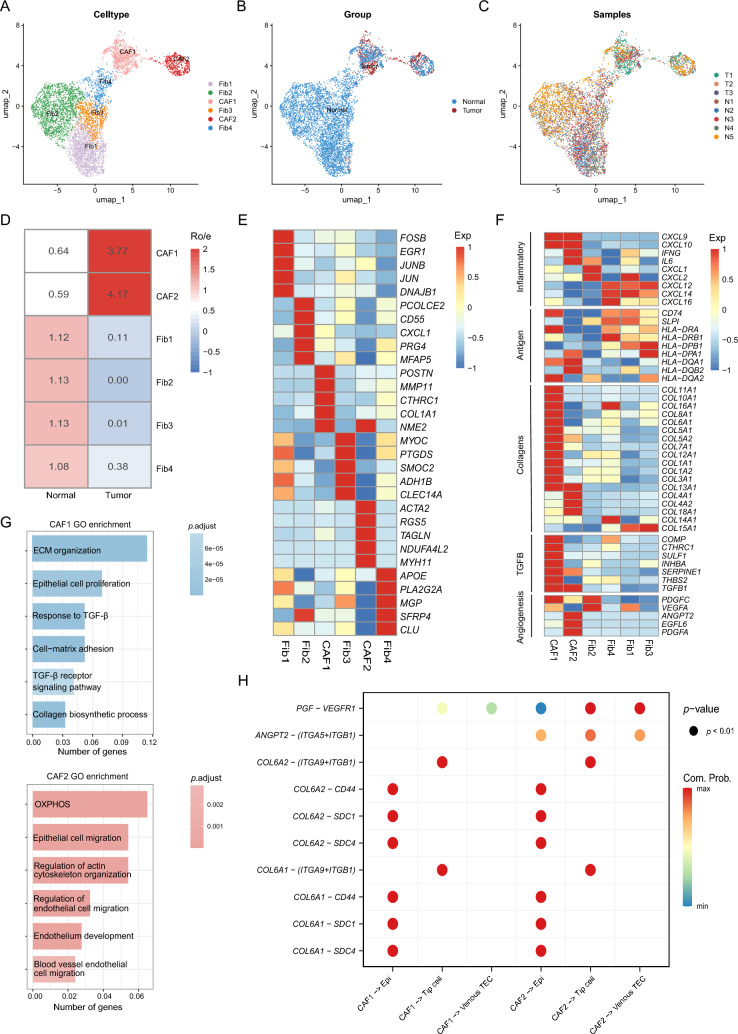
Fibroblast transcriptomic profiling and interactions in HNSCC. **A–C** UMAP of fibroblast subclusters labeled by cell type (**A**), tissue type (**B**) and sample origin (**C**). **D** Tissue preference of each cluster estimated by Ro/e. **E** Heatmap of top 5 DEGs in fibroblast subclusters. Intensity of color indicates average expression of genes. **F** Heatmap showing the scaled expression level of functional gene sets in each fibroblast subcluster. Intensity of color as in (**E**). **G** GO enrichment analysis of DEGs in fibroblast subclusters, color-coded by the* P* value. **H** Bubble plot mapping the interactions between fibroblast cell subtypes, epithelial subtypes, and endothelial subtypes. Dot size indicates the *p* value generated by permutation test and colored by attraction strength levels

Fibroblasts are the most common stromal cell type in the TME, and CAFs play a crucial role in tumor initiation and progression. To understand their heterogeneity, we performed unsupervised clustering and identified six fibroblast populations (Fig. 3A–C). Two subclusters (CAF1 and CAF2) were primarily found in tumor tissues, while the remaining four fibroblast subclusters (Fib1–4) resided mainly in normal tissues (Fig. 3D). The DEGs analysis showed that *POSTN, MMP11,* and *COL1A1* were highly expressed in CAF1, *ACTA2, RGS5*, and *MYH11* in CAF2, *FOSB* and *JUN* in Fib1, *CXCL1* and *PRG4* in Fib2, *MYOC* and *PT*GDS in Fib3, *APOE* and *PLA2G2A* in Fib4 (Fig. 3E and Table S3).

Further analysis revealed functional differences between CAFs and fibroblasts. Fib1, 3, and 4 highly expressed antigen presenting related genes (*HLA-DRA, HLA-DRB1*, and *HLA-DPB1*) and inflammatory cytokines (*CXCL12* and *CXCL14*) while Fib2 highly expressed *CXCL1* and *CXCL2* (Fig. 3F)*.* GO enrichment analysis revealed that all Fib subclusters were enriched with leukocyte development-related pathway and epithelial cell proliferation or migration pathways (Fig. S4A). Compared with fibroblasts, CAF1 displayed enrichment for genes involved in collagens (*COL1A1, COL6A1*, and *COL8A1*) and the transforming growth factor beta (TGF-*β*) pathway (*COMP, CTHCRC1,* and *SULF1*), while CAF2 was enriched in genes associated with angiogenesis (*ANGPT2* and *PDGFA*) and collagen family (C*OL4A1, COL4A2*, and *COL18A1*) (Figs. 3F and S4B). GO enrichment analysis showed that CAF1 was enriched with extracellular matrix organization, epithelial cell proliferation, and TGF-*β* pathway, while CAF2 with regulation of action cytoskeleton organization, epithelial cell migration, and endothelium development, which might promote tumor progression and metastasis (Fig. 3G).

Based on the above tumor-promoting and angiogenesis features of CAF1 and CAF2, we hypothesized that there were complex interactions between CAF and epithelial /endothelial cells. Thus, we performed cell–cell interaction analysis to gain a deeper insight into the regulatory relationships among these subclusters. We found a strong potential interaction with CAFs and epithelial cells via the collagen family (*COL6A1* and *COL6A2*) and their corresponding receptors in epithelial cells (*SDC4, SDC1,* and *CD44*) (Fig. 3H). Moreover, CAF2 was predicted to interact with tumor endothelial cells (TECs) including tip cell and venous TECs via the *ANGPT2*-(*ITGA5* + *ITGB1*) and *PGF*-*VEGFR1* axes, suggesting a role in promoting endothelial cell proliferation and migration (Fig. 3H). Taken together, these findings highlighted the distinct tumor-promoting and angiogenic properties of CAFs and their interactions with epithelial and endothelial cells in promoting tumor progression and metastasis, contrasting with the predominant antigen-presenting and inflammatory roles observed in fibroblasts from normal tissues.

### Tumor endothelial cells and the interactions in the HNSCC ecosystem

**Fig. 4:**
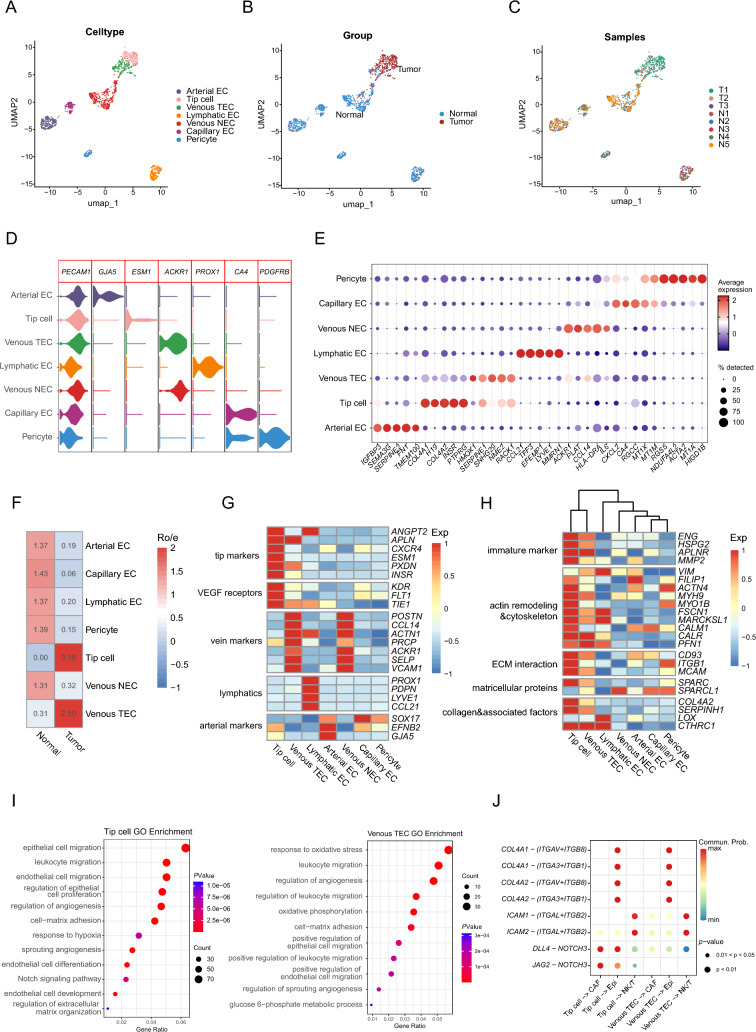
Characteristics of TEC subtypes and interactions in HNSCC. **A–C** UMAP of endothelial cells. Color-coded for cell type (**A**), group (**B**) and sample origin (**C**). **D** Violin plots of select marker gene expression across cell types of endothelial cells from scRNA-seq data. **E** Dot plots show the top 5 DEGs expressed in different endothelial cell clusters. Colors represent the average gene expression levels, and size encodes the proportion of gene-expressing cells. **F** Tissue preference of each cluster estimated by Ro/e. **G** Heatmap of marker gene sets in endothelial cells, with color intensity representing the average expression of genes. **H** Z-scored mean log expression heatmap of functional gene sets across endothelial cell subsets in scRNA-seq data. Intensity of color as in (**G**). **I** Dot plot of GO terms of DEGs in tip cell (left) and venous TEC (right). The dot size indicates enriched gene count, and the color intensity indicates to the *P* value level. **J** Bubble heatmap showing the mean attraction strength for selected ligand–receptor pairs. Dot size indicates *P* value generated and colored by attraction strength levels

Endothelial cells are known to play a critical role in promoting tumorigenesis and development within the tumor ecosystem. Through unsupervised graph-based clustering, seven subclusters were identified with corresponding marker genes, including arterial EC (*GJA5*^+^), tip cell (*ESM1*^+^), venous TEC and venous normal EC (*ACKR1*^+^), lymphatic EC (*PROX1*^+^), capillary EC (*CA4*^+^), and pericyte (*PDGFRB*^+^) (Fig. 4A–E and Table S4). Among these seven subclusters, tip cell and venous TEC mostly resided in malignant tissue, while the others were mainly originated from normal tissue (Fig. 4F).

For TEC subsets, tip cell highly expressed known tip cell markers (*CXCR4*, *ESM1*, *PXDN,* and *INSR*) and vascular endothelial growth factor (VEGF) signaling receptors (*KDR, FLT1*, and *TIE1*), while venous TECs were characterized by vein markers (*POSTN, ACKR1,* and *VCAM1)* (Fig. 4G). Both TEC subclusters displayed an immature phenotype and expressed signature of genes involved in collagen family and actin remodeling and cytoskeleton (Figs. 4H and S5A). Further GO analysis revealed that tip cells were enriched with regulation of sprouting angiogenesis and notch signaling pathways, while venous TEC with angiogenesis and oxidative phosphorylation pathways (Fig. 4I). Meanwhile, both subclusters exhibited enrichment for signaling pathways involved in epithelial and leukocyte migration. Given the potentially complicated communications between TECs and other tumor ecosystem components, we performed cell–cell interaction analysis to evaluate their interactions and potential roles in TME. The analysis suggested that TECs might promote tumor growth and progression through the interaction of *COL4A1*/*COL4A2* with their partner receptors (*ITGAV* + *ITGB8* and *ITGA3* + *ITGB1*) on epithelial cells (Figs. 4J and S5B). In addition, interactions of *JAG2*/*DLL4*-*NOTCH2* were observed between tip cell and epithelial as well as CAF subclusters. Moreover, TECs also interacted with T/NK cells via *ICAM1*/*ICAM2*-(*ITGAL* + *ITGB2*), which might contribute to immune cell infiltration and effective anti-tumor immune response. Collectively, our findings highlight the pivotal role of tip cells and venous TECs in driving tumor progression and the complex crosstalk with other tumor ecosystem components, suggesting a potential double-edged sword effect: facilitating tumor growth while also enhancing the anti-tumor immune response.

### Distinct transcriptomic characteristics of myeloid cells

**Fig. 5:**
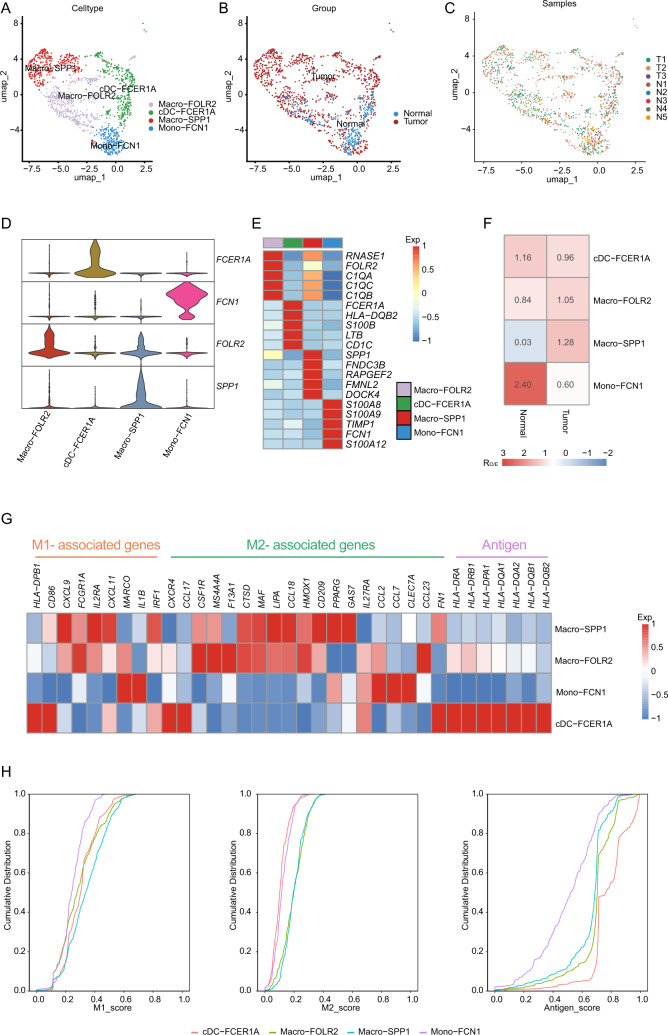
Myeloid cell diversity and functional signatures in HNSCC. **A–C** UMAP representing myeloid cells across the HNSCC, labeled by cell type (**A**), tissue group (**B**), and sample origin (**C**). **D** Violin plots showing the expression of marker genes across four myeloid subclusters. **E** Heatmap of top 5 DEGs in myeloid cell clusters. Intensity of color indicates average expression of genes. **F** Tissue preference of each cluster estimated by Ro/e. **G** Heatmap showing the expression level of M1, M2, and antigen signature genes for different myeloid subclusters. Intensity of color as in (**E**). **H** Cumulative distribution of M1 (left panel), M2 (midst panel), and antigen (right panel) signature scores in each myeloid subtype

To gain a deeper understanding of myeloid cells within the HNSCC tumor ecosystem, we next investigated their composition and heterogeneity. A total of four myeloid subclusters were identified (Fig. 5A–C), including two subtypes of macrophages, Macro-FOLR2 *(FOLR2*^+^) and Macro-SPP1 (*SPP1*^+^), one subcluster of monocyte Mono-FCN1 (*FCN1*^+^), and one subcluster of classic dendritic cells cDC-FCER1A (*CD1C*^+^, *FCER1A*^+^) (Fig. 5D–E, and Table S5). Compared to normal tissues, the tumor group exhibited a higher abundance of Macro-FOLR2, Macro-SPP1 and cDC-FCER1A, with a lower proportion of Mono-FCN1 (Fig. 5F). To further explore the role of these clusters in the anti-tumor immune response, we further evaluated the M1, M2, and antigen-presenting features and gene sets scores among the myeloid cell subclusters, which revealed the genes associated with antigen presentation were strongly expressed in cDC-FCER1A (Fig. 5G, H). Furthermore, both M1- and M2-associated genes were highly expressed in Macro-FOLR2, Macro-SPP1, and Mono-FCN1. However, the expression profiles of M1- and M2-related genes differed between these subclusters. For example, the chemotaxis genes such as *CCL23* were enriched in Macro-FOLR2, while *CCL7* was highly expressed in Mono-FCN1 [23–25]. These findings suggest that macrophages and monocytes exhibited characteristics of both anti-inflammatory and pro-inflammatory phenotypes.

### Characteristics of lymphocyte subclusters and their correlation with patient survival

**Fig. 6:**
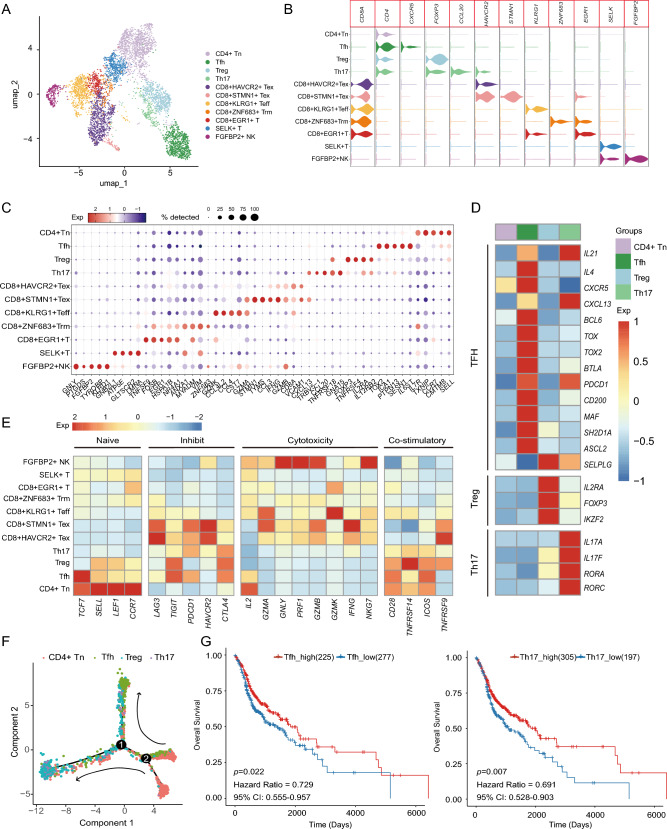
Characteristics of T/NK cell clusters and prognostic correlation in HNSCC. **A** UMAP plot showing 11 T/NK subclusters colored by cell clusters. **B** Violin plots showing the marker genes expression across the T/NK cell clusters. **C** Bubble heatmap showing top 5 marker genes across T/NK cell subclusters, with the size of dot size proportional to the percentage of cells expressing the gene, and color intensity based on z score normalized expression levels. **D** Gene expression levels of feature genes in the CD4^+^ T cell subclusters. Intensity of color indicates average expression of genes. **E** Heatmap showing the expression of functional gene sets in T/NK cell clusters. Intensity of color as in** D**. **F** The trajectory analysis of the CD4^+^ T cell subclusters in all samples, colored by cell type. **G** Overall survival curves of 502 HNSCC patients, stratified by high or low enrichment of Tfh cells (left panel) and Th17 cells (right panel). The *p* values correspond to the log-rank tests

Based on the high-resolution T/NK cell map in HNSCC, we identified four distinct CD4^+^ T cell clusters including naïve CD4^+^ T (CD4^+^ Tn) cells (*SELL*^+^), Tfh cells (*CXCR5*^+^), Th17 cells (*CCL20*^+^), and regulatory T (Treg) cells (*FOXP3*^+^). We also identified five CD8^+^ T cell clusters consisting of two exhausted T cells, CD8^+^HAVCR2^+^ Tex *(HAVCR2*^+^) and CD8^+^STMN1^+^ Tex (*STMN1*^+^), as well as CD8^+^KLRG1^+^ effector T (Teff) (*KLRG1*^+^), CD8^+^ZNF683^+^ tissue-resident memory (Trm) (*ZNF683*^+^), and CD8^+^ EGR1^+^ T cell subsets. Additionally, we identified SELK^+^ T and FGFBP2^+^ NK cells (Fig. 6A–C, Fig. S6A, B and Table S6). Comparing the proportions of T/NK cells between normal and tumor groups revealed an enrichment of the four CD4^+^ T cell clusters, two exhausted CD8^+^ T cell subsets, and CD8^+^ZNF683^+^ Trm cells in the tumor group (Fig. S6C).

Distinct transcriptional characteristics and gene expression patterns were observed in these CD4^+^ T cell subsets, including classical naïve markers *(LEF1, TCF7, SELL*, and *CCR7*) in CD4^+^ Tn, Tfh-signature genes (*CXCR5, CXCL13, BCL6*, and *TOX*) in Tfh, inflammatory cytokines (*IL17A* and *IL17F*) in Th17, inhibitory genes (*IL2RA, FOXP3* and *IKZF2*) in Treg (Fig. 6D–E). Consistent with these findings, CD4^+^ Tn cells were characterized with the highest naïve signature score among the T/NK subclusters, while Treg cells simultaneously exhibited the highest costimulatory gene (*TNFRSF9* and *TNFRSF14*) signature scores (Figs. 6E and S6D). For CD8^+^ T and NK subclusters, the two Tex clusters (CD8^+^HAVCR2^+^ Tex and CD8^+^STMN1^+^ Tex) highly exhibited both cytotoxic (*GZMA* and *GZMB)* and inhibitory (*LAG3* and *HAVCR2*) gene signature scores, while CD8^+^STMN1^+^ Tex also expressed cell cycle-related genes like *STMN1* and *TYMS* (Fig. 6C, E). Moreover, the Teff cluster (CD8^+^KLRG1^+^ Teff) was mainly characterized by cytotoxic effectors (*GZMA* and *GZMK*) (Fig. 6E). Similarly, FGFBP2^+^ NK cells also highly expressed markers like *GNLY*, *NKG7*, and *PRF1*, indicating a strong anti-tumor immune response (Fig. 6E).

To elucidate the lineage development between diverse CD4^+^ T cells, we performed trajectory analysis and found that naïve CD4^+^ T cells and partial Tfh cells underwent divergent differentiation into two branches, one dominated by Th17 and Treg cells, and the other by Tfh cells (Fig. 6F). Further GO analysis revealed that Tfh cells were enriched with lymphocyte proliferation, B and T cell activation, and leukocyte cell–cell adhesion pathways, while Th17 cells were with B, NK, and T cell activation, DC differentiation and activation, and interferon-gamma production pathways (Fig. S7A). Based on these functional profiles, we hypothesized that Tfh and Th17 populations could promote the anti-tumor immune response in HNSCC patients, hence the relative infiltration of these cells might be correlated with clinical outcome. Therefore, we analyzed bulk RNA-seq data from HNSCC patients (*n* = 502) and found that high frequencies of Tfh or Th17 cells were significantly associated with improved patients’ overall survival and progression-free survival (all *p* < 0.05) (Figs. 6G and S7B). These findings suggest that a higher infiltration of Tfh and Th17 cells predicts better outcomes for HNSCC patients. Therefore, our analysis revealed that in HNSCC, the enhanced infiltration and presence of Tfh and Th17 cells were significantly correlated with improved patient survival, underscoring the importance of these subclusters in promoting effective anti-tumor immunity.

In terms B and plasma cells, which were almost derived from tumor group, four clusters were distinguished to investigate the heterogeneity and roles of B and plasma cells in HNSCC (Fig. S8A). On the basis of the transcriptomic profiles, we identified three B subclusters including TCL1A^+^ naïve B cell (*TCL1A* and *FCER2*), CD27^+^ memory B (Bm) cells (*CD27* and *ITGB1*), and DUSP4^+^ atypical memory (Atm) B cells (*DUSP4* and *FCRL4*). We also uncovered the MZB1^+^ plasma cells (PC), highly expressing *MZB1* and *IGHG1* (Fig. S8B and C; Table S7). Further functional analyses suggested that three B subclusters were particularly concentrated in the lymphocyte proliferation and activation pathway, while the MZB1^+^ PC was enriched in immunoglobulin mediated immune response and complement activation pathways, implying the distinct anti-tumor functions (Fig. S8D). Based on the pro-inflammatory characteristics of B and plasma cells, we further explored the prognostic significance of these subclusters and performed survival analysis in the TCGA-HNSCC cohort. We found that the frequency of TCL1A^+^ naïve B, DUSP4^+^ Atm, and MZB1^+^ PC were positively correlated with survival (all *p* < 0.05; Fig. S8E). Together, these data showed that B and plasma cells promoted anti-tumor immune response and were positively associated with patient survival with HNSCC.

## Discussion

The heterogeneous cellular composition and the complex interplay within the tumor ecosystem play critical roles in the tumor pathogenesis and invasiveness. Here, we have leveraged the advantages of scRNA-seq approach to depict a detailed landscape of HNSCC ecosystem, identifying the immune features of cell populations and prognostic factors. Our analysis revealed three epithelial subclusters with immune-related features, inducing T cell, DC, and monocyte infiltrated into the TME. Additionally, we found that Tfh and Th17 cells might promote the anti-tumor response through activating B and T cells, and their presence were significantly correlated with better outcomes of HNSCC patients. Furthermore, Fib1–4 mainly from normal tissues exhibited diverse inflammatory roles, while CAF1 and CAF2 displayed tumor-promoting characteristics through interactions with epithelial and endothelial cells. We also highlighted the dual role of tip cells and venous TECs, where they promote tumor progression while also potentially strengthening the immune response. Taken together, these results underscored the importance of immune features within the tumor ecosystem and provided prognostic significance of CD4^+^ T cells for HNSCC patients.

In HNSCC, the composition and transcriptional characteristics of epithelial cells exhibited significant intra-tumor heterogeneity. Among 11 epithelial subclusters, Epi0, 9, and 10 displayed strong immune-related features. These clusters were characterized by high expression of chemokine family genes, suggesting a potential role in promoting immune cell infiltration. Similar findings of epithelial-immune dual features in malignant cells have been reported in nasopharyngeal carcinoma and cervical cancer [10, 26]. In addition, Dai et al. performed scRNA-seq analysis on HNSCC samples and divided epithelial cells into three intrinsic consensus molecular subtypes [13]. One of these subtypes, characterized by an immune-enriched profile with abundant infiltrating immune cells (T cells, B cells, and NK cells) in TCGA-HNSC bulk samples, also displayed the best prognosis of HNSCC patients compared to other subtypes. We further focused on the transcriptional profiles of the three pro-inflammatory clusters and their potential crosstalk with immune cells. For instance, Epi9 might play a role in facilitating the migration of T cell, DC, and monocyte into TME through *CXCL16*-*CXCR6* and *CXCL17*-*GPR35* crosstalk, which have not been reported before. Notably, Epi0 displayed enrichment for interferon-related response and might induce CD8^+^ T cells and CD4^+^ naïve T infiltration via *CXCL9*/*11*-*CXCR3*/*DPP4* and *CXCL14*-*CXCR4*, suggesting a robust pro-inflammatory immune response. Further correlation analysis supported the induction of immune cell infiltration and *CXCL9, CXCL11, CXCL14*, *CXCL17, CXCR3, CXCR4*, *CXCR6,* and *GPR35* predicted better survival of HNSCC patients. These findings revealed the epithelial subclusters with immune-related characteristics might promote immune cell infiltration and provide prognostic biomarkers for HNSCC patients.

Within the fibroblast clusters, we revealed the distinct characteristics of two main subclusters resident in tumor tissues, CAF1 and CAF2, and extensively investigated the crosstalk between these CAF subclusters and Epi as well as EC subclusters. CAF1 highly expressed genes associated with TGF-*β* pathway, which not only contributes to CAF activation and formation [27], but also plays a crucial role in promoting malignant biological behaviors of tumor cells, including proliferation, invasion, metastasis, and stemness [28–31]. CAF2, on the other hand, was predicted to interact with endothelial cells through the *PGF*-*VEGFR1* signaling axis. PGF, a well-known vascular growth factor, is reported to be involved in angiogenesis [32]. Upon binding to VEGFR1, PGF activates signaling pathways that promote blood vessel formation [33]. Based on our findings, it is highly conceivable that CAF2 secretes PGF, which binds to VEGFR1 on endothelial cells. This interaction triggers a cascade of signaling events, leading to endothelial cell proliferation, migration, and lumen formation, ultimately promoting the formation of new blood vessels. Overall, these findings provide significant insights into the mechanisms of tumor angiogenesis and hold promise for developing anti-tumor therapeutic strategies for HNSCC.

TECs, a key component of the TME, play a significant role in promoting tumor growth and invasion through diverse mechanisms. Our study revealed two main TEC subclusters found in human HNSCC: tip cells and venous TECs. The immature phenotype observed in both subclusters indicates the presence of an immature and dysfunctional vascular network within HNSCC. This dysfunctional network may fail to provide adequate oxygen and nutrients to the tumor, leading to a hypoxic TME and hindering immune cell infiltration [34]. Immature TECs have also been significantly associated with decreased survival in patients with non-small cell lung cancer [21]. Moreover, the interactions of TEC subclusters with other tumor ecosystem components exhibited a double-edged sword effect. While they could promote tumor progression, they also enhance the anti-tumor immune response. For instance, tip cells might interact with CAFs and epithelial cells through *JAG2*/*DLL4*-*NOTCH2* axes. The notch signaling pathway has been reported to be involved in tumor formation and proliferation through activating NF-κB and regulating progenitor tumor cell differentiation in esophagus cancer [35]. On the other hand, TECs also might contribute to lymphocyte migration into the tumor via *ICAM1*/*ICAM2*-(*ITGAL* + *ITGB2*) interactions with T/NK cells. This is supported by studies showing that the high densities of tumor-associated high endothelial venules expressing *ICAM1* in breast cancer have been demonstrated to be correlated with the increase of T cell infiltration and cytotoxicity, as measured by flow cytometry and PCR analyses [36]. Additionally, the interaction between *ICAM1* and *ITGAL* could also play a critical role in the transendothelial migration of leucocytes and lymphocyte activation [37].

The plasticity of tumor-associated macrophages (TAMs), allowing them to rapidly and reversibly shift among distinct functional phenotypes in response to signal changes within the TME, extends beyond the conventional M1/M2 polarization theory [9, 38]. Our study identified two macrophage subclusters, Macro-FOLR2 and Macro-SPP1, which co-express markers of both M1 and M2 phenotypes. This finding aligns with observations in breast cancer, which also revealed that all TAM populations highly expressed both M1 and M2 gene signatures [39]. Therefore, the traditional M1/M2 dichotomy is not sufficient to accurately classify TAMs. Currently, therapeutic strategies targeting TAMs are being developed and show promising preclinical and clinical outcomes. These strategies include reprogramming TAMs from a pro-tumor M2 phenotype to an anti-tumor M1 phenotype, inhibiting TAMs recruitment, and depleting TAMs. A comprehensive understanding of the phenotypic and functional characteristics of TAMs is crucial for developing macrophage-targeted therapies and improving the prognosis of HNSCC.

Among the T/NK subclusters, two CD4^+^ T cell clusters (Tfh and Th17) were found to be significantly associated with better survival in HNSCC patients. However, Tfh and Th17 cells did not show high expression of cytotoxic-related effectors like granzyme B or perforin, indicating that their anti-tumor immunity may not be direct. Further analysis showed that Tfh cells, characterized by high expression of *CXCR5, CXCL13*, *BCL6,* and *TOX*, were enriched with lymphocyte proliferation, B and T cell activation, and leukocyte cell–cell adhesion pathways. Supporting this, recent studies confirmed that the interactions between Tfh and B cells in germinal center are essential for driving effective functions of tumor-infiltrating CD8^+^ T cells and tumor control [40]. Additionally, the CXCL13 produced by Tfh cells might also contribute to the formation of tertiary lymphoid structures within tumors [41]. In several malignant tumors, B cell infiltration into the TME and the formation of tertiary lymphoid structures have been reported to be correlated with better patient survival [42, 43]. In terms of Th17 cells, characterized by the pro-inflammatory cytokine IL-17, enriched with B, NK and T cell activation, DC differentiation and activation, and interferon-gamma production pathways in this study. Moreover, Th17 cells have been shown to induce robust activation of CD8^+^ T cells [44] and could also stimulate the production of the chemokine CCL20 by tumor tissues, promoting the infiltration of DC in a *CCL20*-*CCR6* dependent manner[45]. As expected, we found that the higher abundances of Tfh and Th17 cells predicted better survival for HNSCC patients, demonstrating the critical role of Tfh and Th17 infiltration in the anti-tumor immune response. It is generally accepted that T helper cells could be differentiated from naïve CD4^+^ T cells, facilitated by antigen presentation and cytokine secretion from DC cells [46, 47]. Similarly, we also revealed that Tfh and Th17 cells might be differentiated from CD4^+^ Tn cells. Meanwhile, it was found that Epi0 may chemotaxis CD4^+^ Tn into the TME via *CXCL14*-*CXCR4*, which subsequently differentiated into Tfh and Th17, inducing the formation of a tumor inhibitory immune microenvironment. Moreover, we also found that Th17 cell infiltrated might be induced into TME by the interaction with Epi10 through *CXCL16*-*CXCR6*. In conclusion, Tfh and Th17 cells might indirectly promote the anti-tumor immune response by facilitating the recruitment and activation of other immune cells. These findings suggest their potential utility as prognostic factors for risk stratification of HNSCC patients.

Recently, the anti-tumor properties of B and plasma cells have been increasingly recognized [48, 49]. This study’s data highlighted the heterogeneity of B and plasma subclusters and revealed favorable prognostic factors in patients with HNSCC. We were able to recapitulate the heterogeneity of B and plasma cells (naïve, Bm, Atm, and PC) as well as to show the differential transcriptomic profiles. As previously reported, with the anti-tumor capabilities of antigen presentation and antibodies producing against tumor-associated antigens, B and plasma cells may closely interact with T cells and predict hot immune microenvironment and better survival in several cancers [50–52]. We also found the activation of immune response in these subclusters and revealed TCL1A^+^ naïve B, DUSP4^+^ Atm, and MZB1^+^ PC as favorable prognostic factors for patients with HNSCC.

Although our study has provided a detailed landscape of the HNSCC ecosystem, it is important to acknowledge several limitations. Firstly, the sample size in our study was relatively small, and larger-scale studies are required to replicate and validate our findings. Secondly, additional spatial information is needed to understand the true localization of the identified cells in HNSCC. Lastly, inferring direct cell–cell interactions and signaling pathways solely from scRNA-seq data can be challenging, meaning that additional characterization of crosstalk between different cells in the tumor ecosystem of HNSCC should be performed. Therefore, further complementary approaches, such as multi-omics approaches, multiplexed protein techniques, and functional assays will likely be needed to corroborate and validate our scRNA-seq findings.

## Conclusion

In conclusion, this study harnessed scRNA-seq to comprehensively investigate the HNSCC ecosystem, revealing the immune-related features of diverse cell populations and identifying Tfh and Th17 cells as potential prognostic factors. These findings shed light on the underlying regulation of anti-tumor immune response and potentially guide the development of novel therapeutic targets for future HNSCC treatment strategies.

## Supplementary Information

Below is the link to the electronic supplementary material.

Supplementary file1 (DOCX 2627 kb)

Supplementary file2 (XLSX 1571 kb)

## Data Availability

The scRNA-seq data in this study have been deposited in the OMIX, China National Center for Bioinformation (https://ngdc.cncb.ac.cn/omix: accession no. OMIX006628).

## Abbreviations

Atm: Atypical memory B
Bm: Memory B
CAFs: Cancer-associated fibroblasts
DC: Dendritic cell
DEGs: Differentially expressed genes
Epi: Epithelial
Fib: Fibroblast
GO: Gene ontology
GSVA: Gene set variation analysis
HPV: Human papillomavirus
HNSCC: Head and neck squamous cell carcinoma
PC: Plasma cells
scRNA‐seq: Single‐cell RNA sequencing
TME: Tumor microenvironment
Tfh: Follicular helper T
Th17: T helper 17
TAMs: Tumor-associated macrophages
TECs: Tumor endothelial cells
UMIs: Unique molecular identifiers
