# Supplementary material for: Highlighting immune features of the tumor ecosystem and prognostic value of Tfh and Th17 cell infiltration in head and neck squamous cell carcinoma by single-cell RNA-seq
Source: Cancer Immunol Immunother. 2024 Aug 2;73(10):187. doi: 10.1007/s00262-024-03767-6 (PMC11297013; doi:10.1007/s00262-024-03767-6)
Supplement: Supplementary file 1 — Supplementary file1 (DOCX 2627 kb) [file 262_2024_3767_MOESM1_ESM.docx]

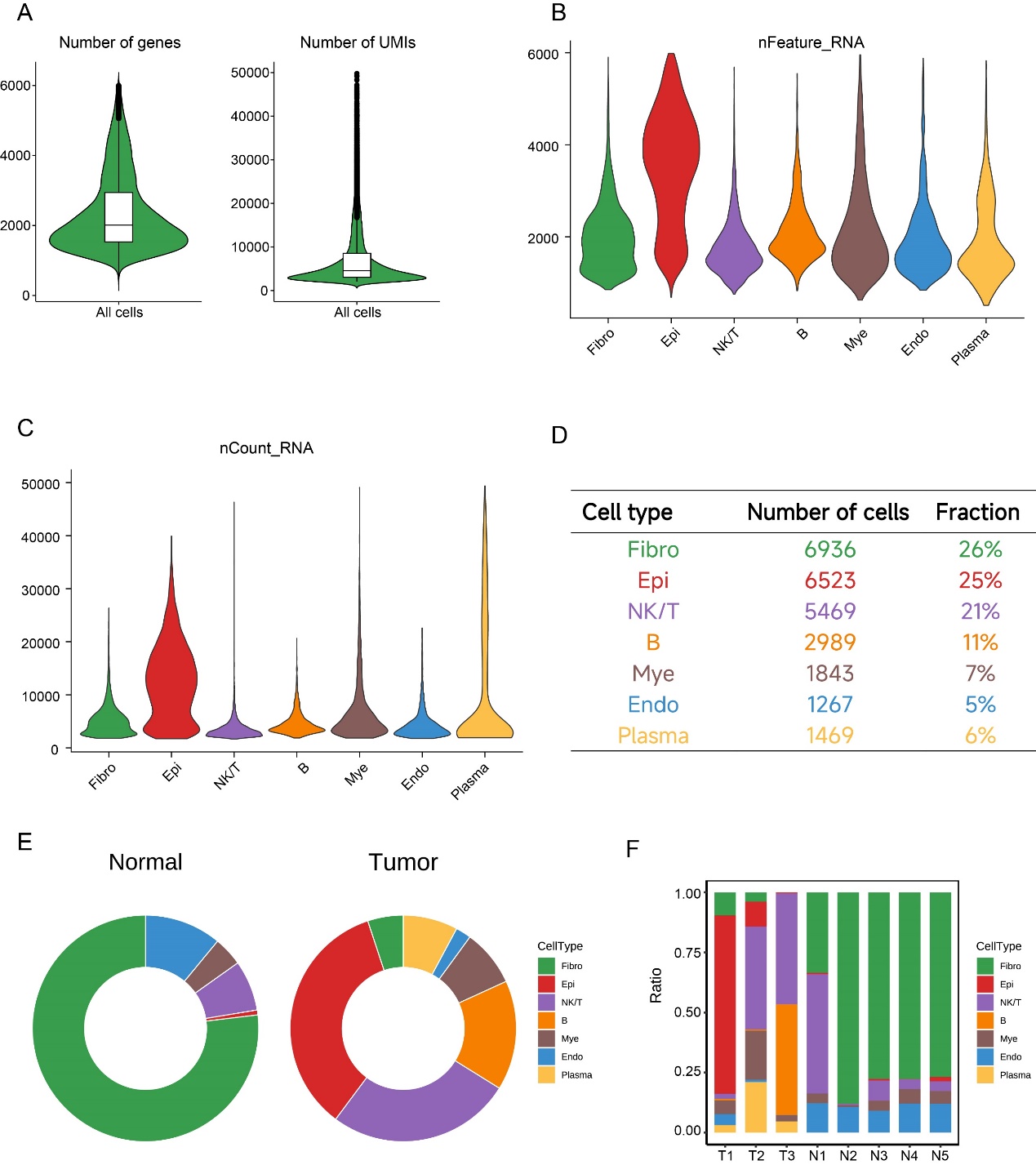


**Fig. S1 scRNA-seq data quality control.**

**A** Violin plots of genes and unique molecular identifiers (UMIs) counts of 26,496 cells.

**B-C** Violin plots of genes (B) and UMI counts (C) per cell type.

**D** The table showing the number and relative proportions of each cell type.

**E** Cell compositions shown according to the tissue type, colored by cell types.

**F** Proportions of each cell type in each sample, colored by cell types.


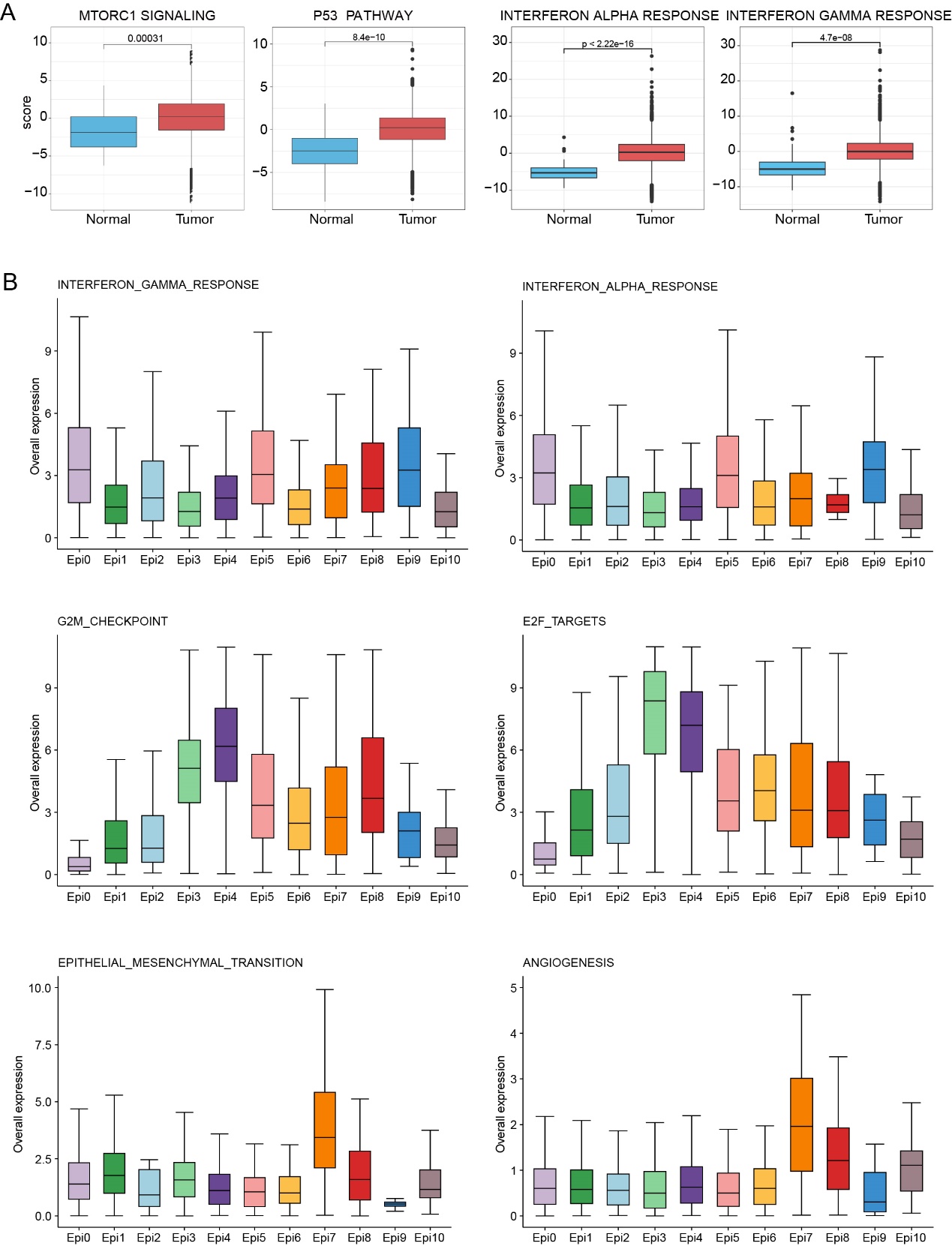


**Fig. S2 Tumor hallmark analysis of** **epithelial cells.**

**A** Boxplots showing the distribution of gene set variation analysis (GSVA) scores for tumor hallmark pathways across normal and tumor-derived epithelial cells. Statistical significance was evaluated by t-tests.

**B** Tumor hallmarks of interferon-alpha response, interferon-gamma response, G2M checkpoint, E2F targets pathways, epithelial–mesenchymal transition and angiogenesis among the epithelial subclusters.


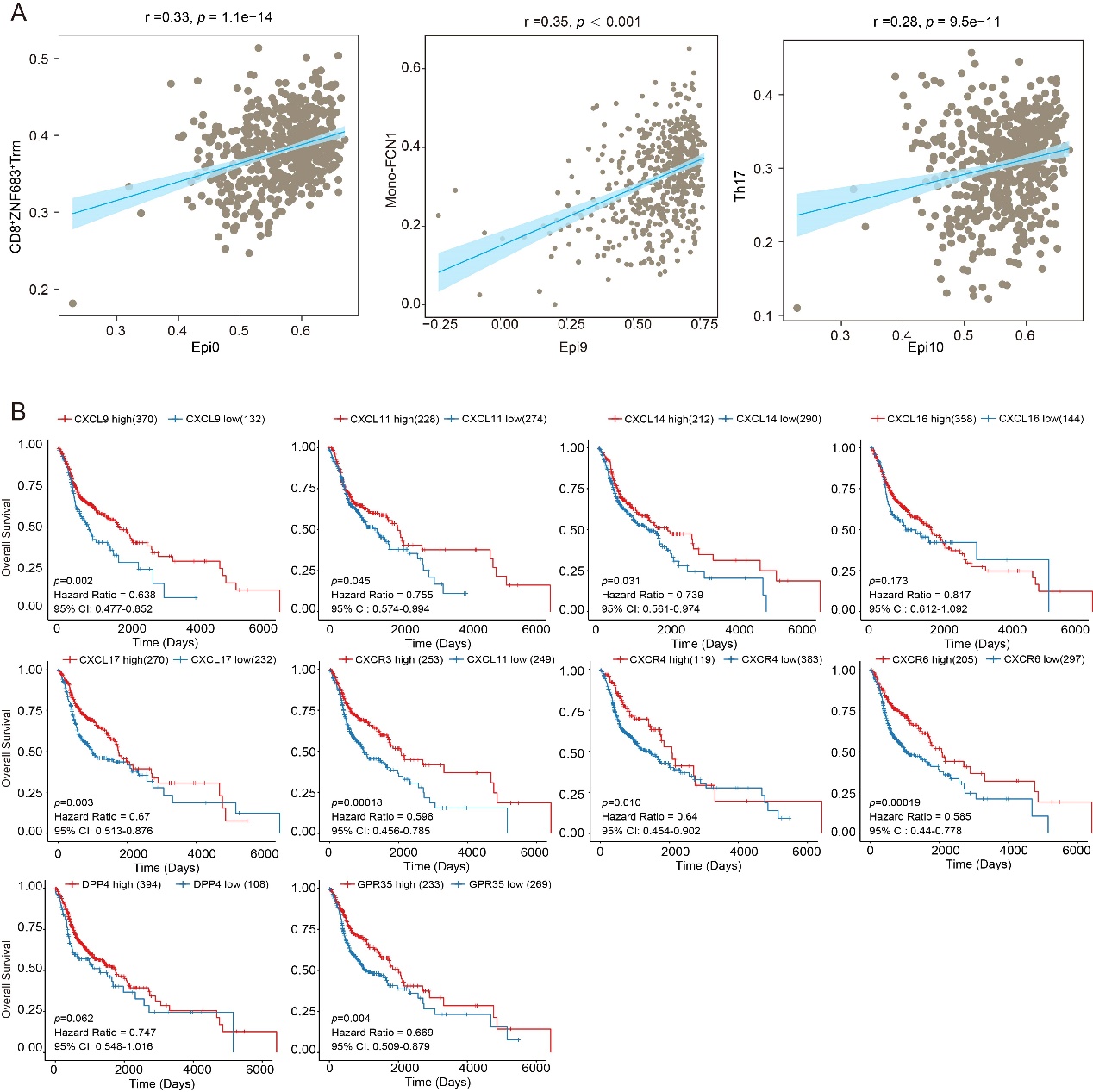


**Fig. S3 Correlation between** **epithelial subclusters and immune cells and the prognostic value of ligand–receptor pairs**

**A** Plots shows the correlation between the proportion of Epi0, 9, 10 and immune cells in TCGA-HNSCC data.

**B** Overall survival curves of 502 HNSCC patients, stratified by high or low of the ligand–receptor pairs expression between epithelial subclusters and immune cells. The *p* values correspond to the log-rank tests.


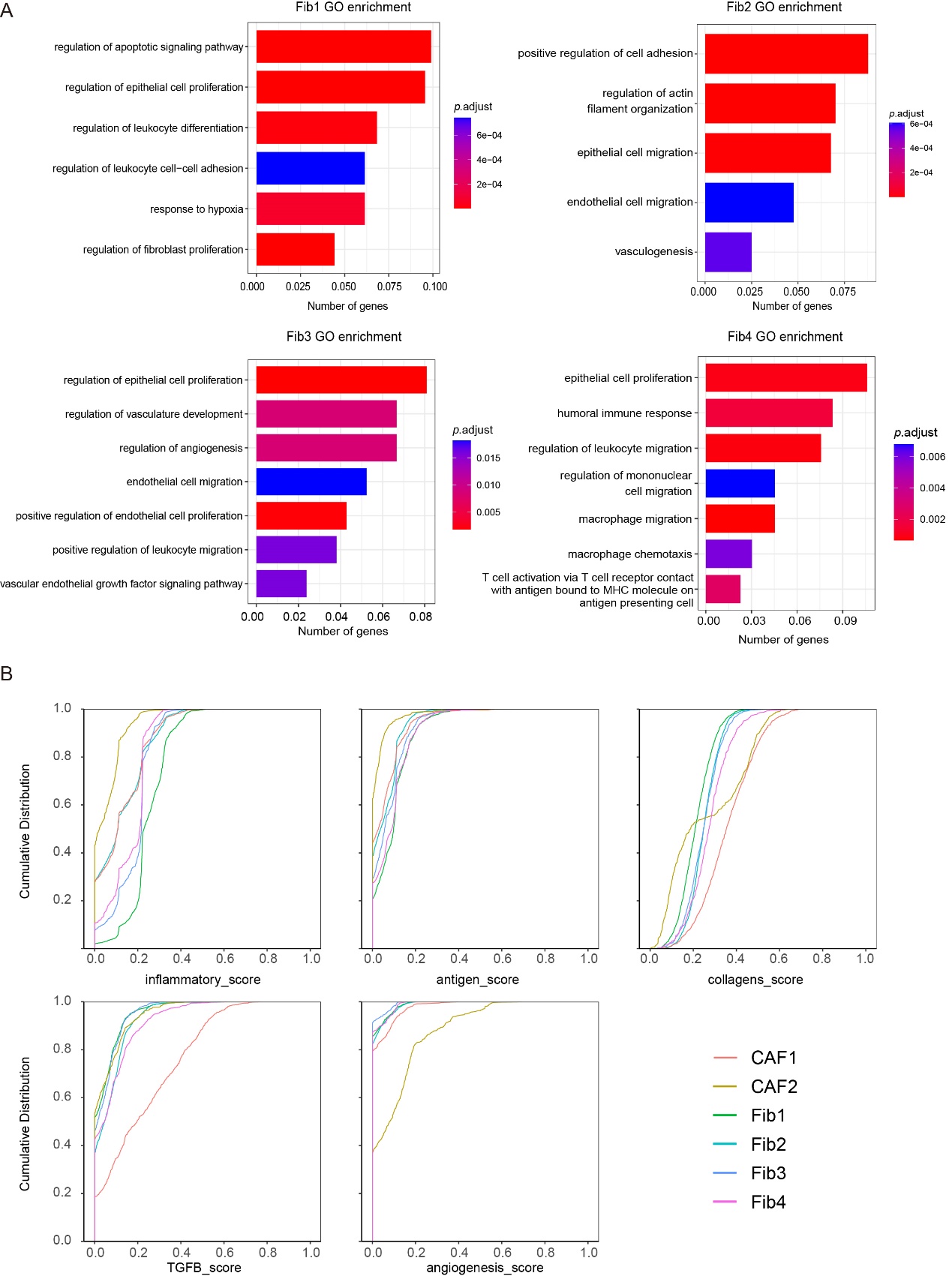


**Fig. S4** **Heterogeneity within the CAF and Fib subclusters.**

**A** Gene Ontology (GO) term analyses of differentially expressed genes (DEGs) in fibroblast subclusters.

**B** Cumulative distribution function showing the inflammatory, antigen, collagens, TGF-β and angiogenesis signature scores in each fibroblast subtype.


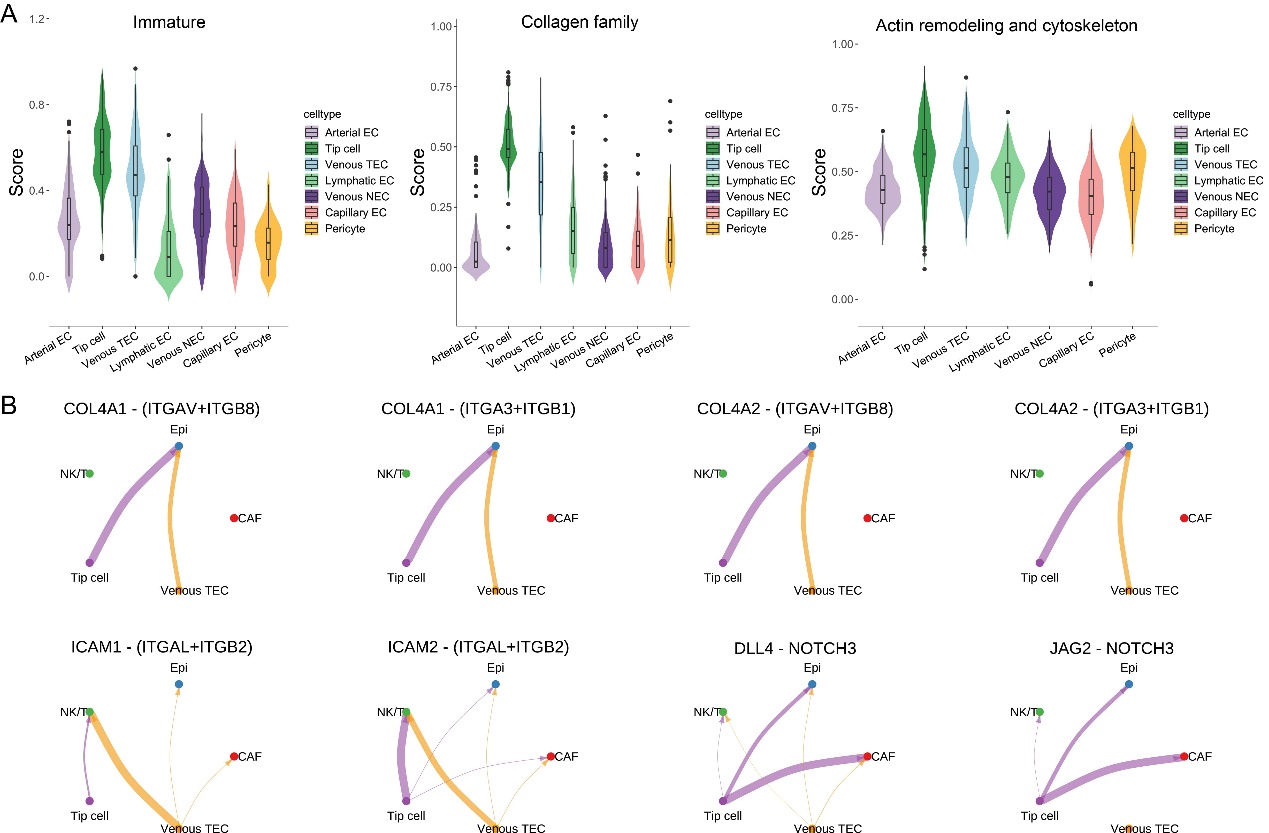


**Fig. S5 Detailed Characterization of TEC clusters.**

**A** Signature gene set scores of the EC subclusters, colored by cell cluster.

**B** Representative circle plots showing detail interactions between TEC subsets and other components within the tumor ecosystem.


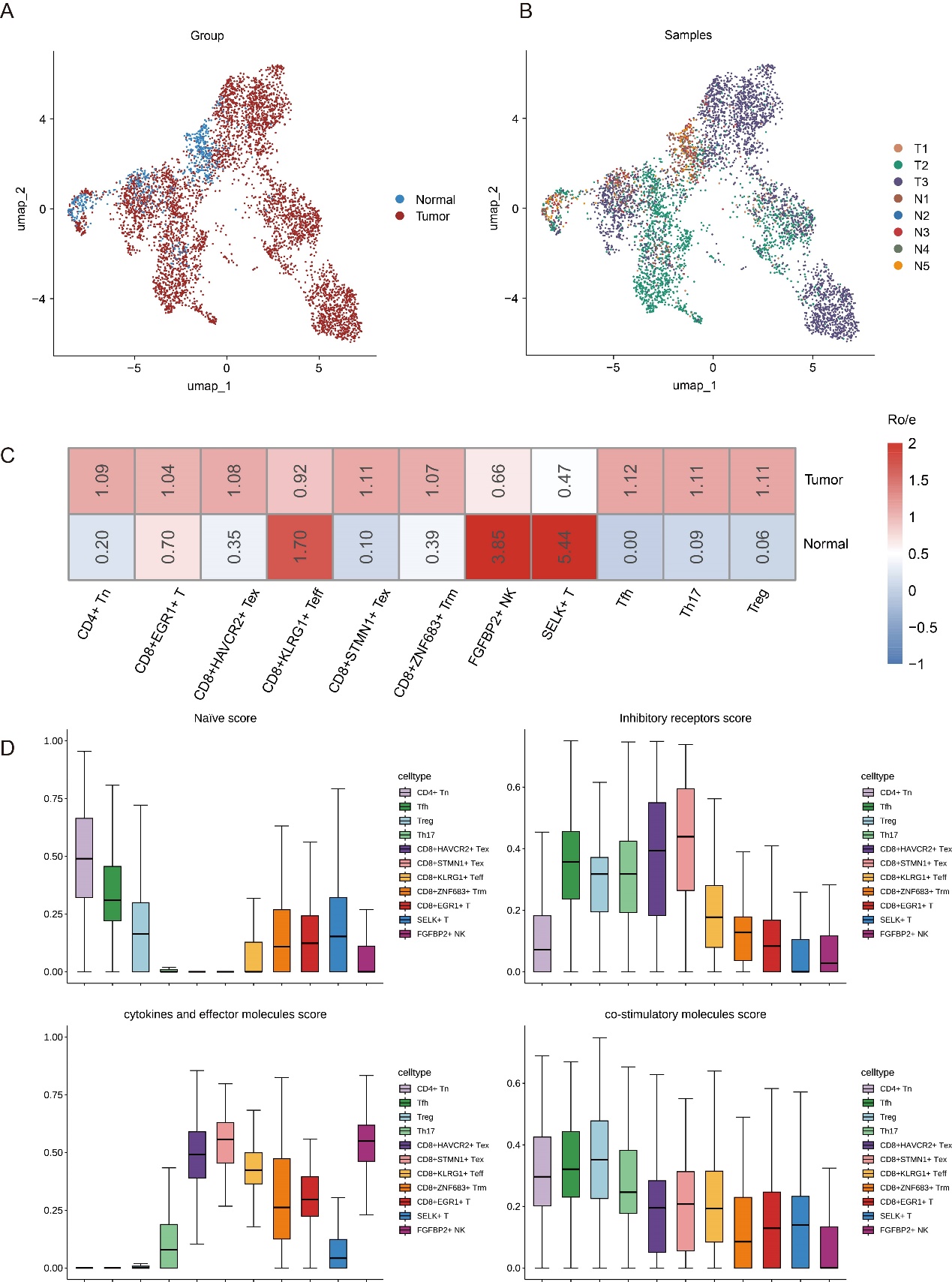


**Fig. S6 The relative proportion and functional analysis of T/NK subclusters.**

**A-B** Uniform manifold approximation and projection (UMAP) of T/NK subclusters labeled by tissue type (**A**) and sample (**B**)

**C** Tissue preference of each cluster estimated by Ro/e.

**D** Box-plots showing naïve, co-stimulatory, cytotoxicity and inhibit scores within each of the T/NK cell subclusters.


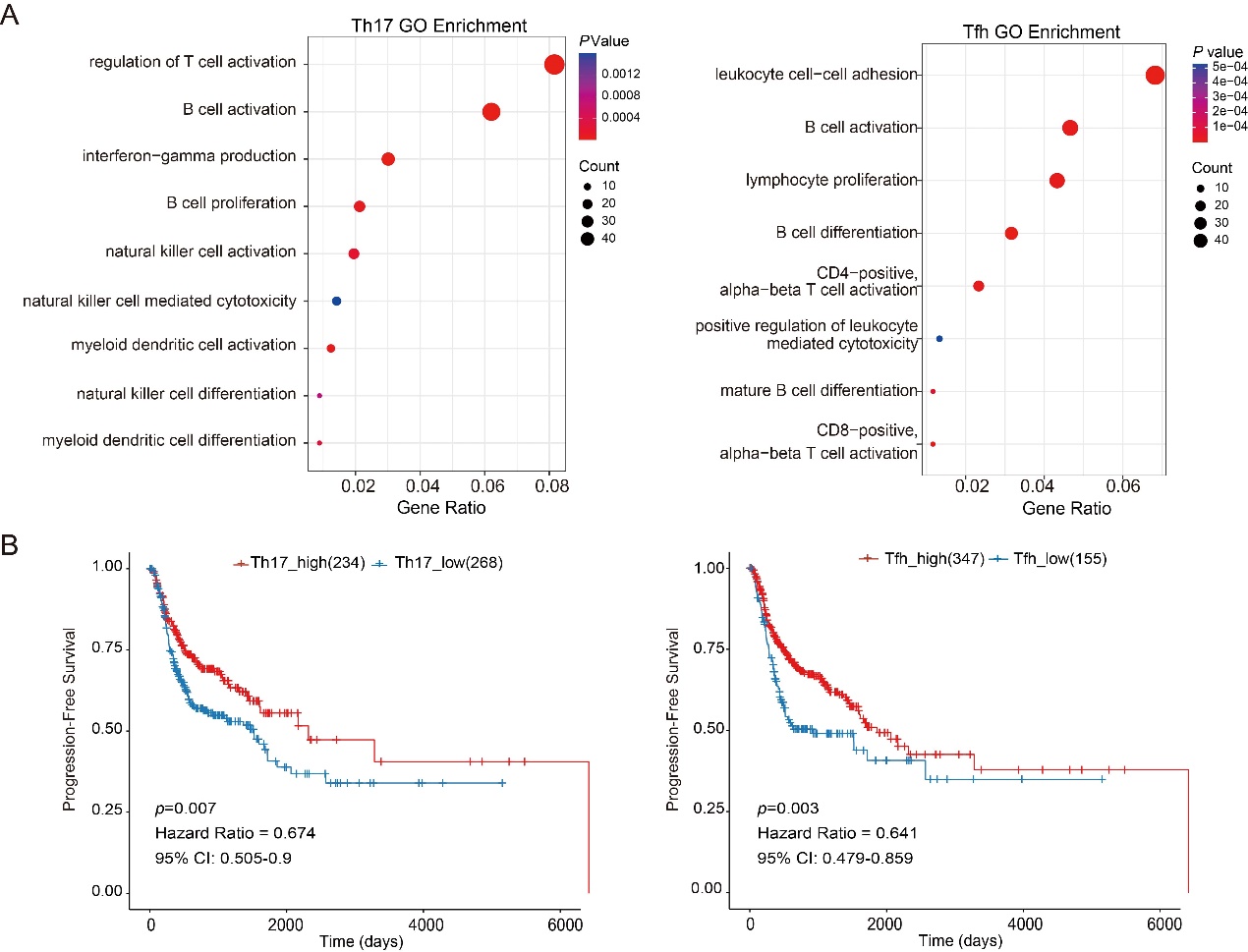


**Fig. S7 The characteristics and clinical implication of Th17 and Tfh in HNSCC.**

**A** Dot plots of GO terms of DEGs in Th17 and Tfh.

**B** The progression-free survival curves of HNSCC patients, grouped by high or low enrichment of Th17 cells (left panel) and Tfh cells (right panel). The *p* values correspond to the log-rank tests.


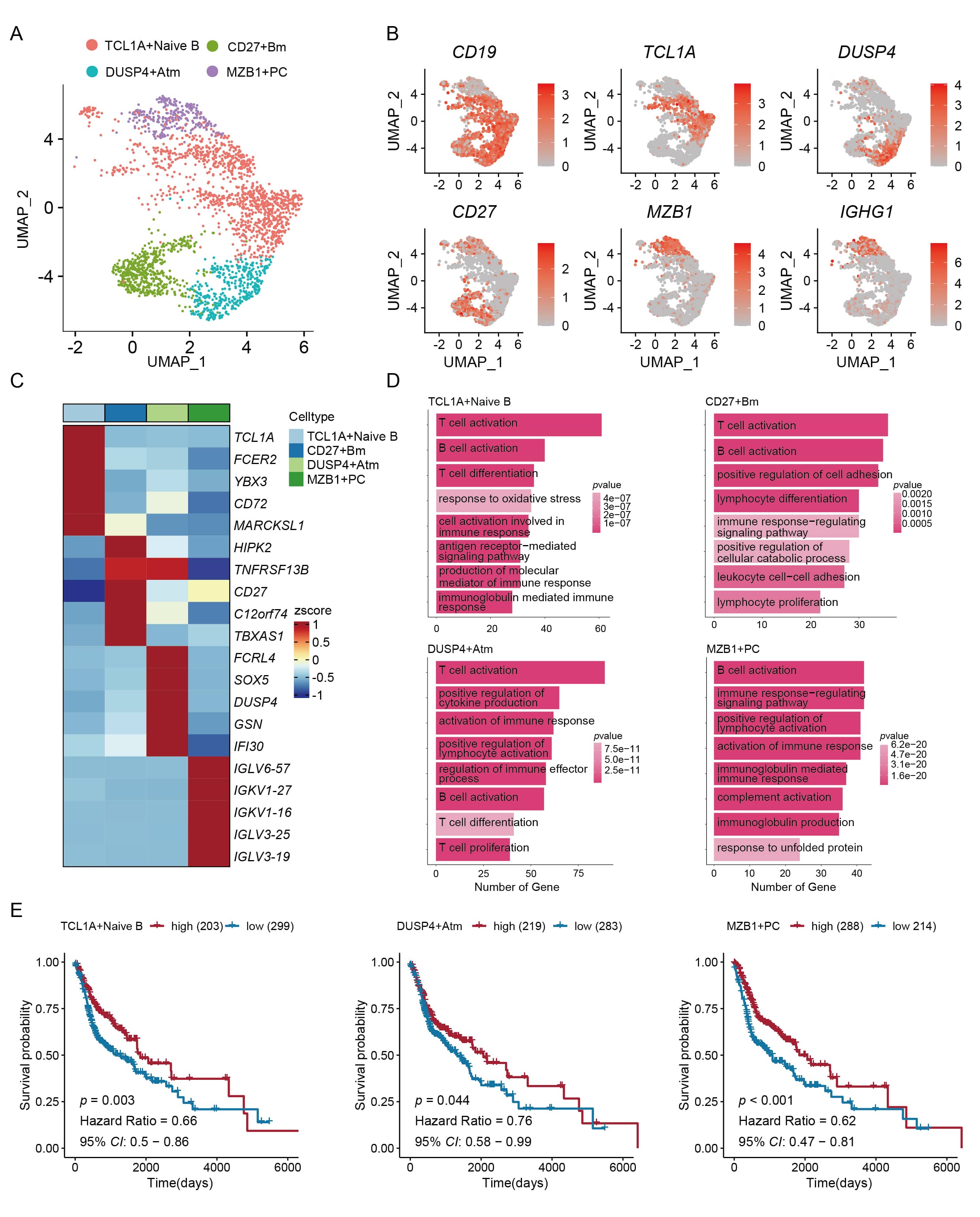


**Fig. S8 The pro-inflammatory roles and prognostic significance of B and plasma cells in HNSCC**

**A** UMAP plot identifying four B and plasma subclusters, with each cell represented by a dot, color-coded by cell type.

**B** UMAP plots showing the selected marker genes expression across clusters. Intensity of color indicates normalized gene expression.

**C** Heatmap showing top five DEGs of cell subclusters, with intensity of color representing average expression of genes.

**D** GO terms showing the selected signaling pathways enriched in B and plasma subclusters.

**E** Kaplan-Meier overall survival analysis of TCGA HNSCC patients stratified by high or low enrichment of subclusters. The *p* value of two-sided log-rank test is shown.
